# Water body type and group size affect the flight initiation distance of European waterbirds

**DOI:** 10.1371/journal.pone.0219845

**Published:** 2019-07-16

**Authors:** Martin Mayer, Daniel Natusch, Shane Frank

**Affiliations:** 1 Department of Bioscience, Aarhus University, Rønde, Denmark; 2 Department of Natural Sciences and Environmental Health, University of South-Eastern Norway, Bø i Telemark, Norway; 3 Department of Biological Sciences, Macquarie University, North Ryde, New South Wales, Australia; Liverpool John Moores University, UNITED KINGDOM

## Abstract

Human encroachment on nature grows constantly, increasing human-wildlife interactions. Flight initiation distance (FID, the distance at which animals flee from an approaching threat) is often used to measure antipredator behaviour and establish buffer zones to reduce human impact on wildlife. In this study, we approached 10 waterbird species on larger lakes and narrower rivers using a motorboat. We investigated whether water body type, season (winter/spring), approach starting distance, species body mass, and group size influenced bird FID. Average bird FID was 145 ± 92 m and differed between species. In general, FID of all species was larger on lakes than rivers and increased with increasing group size and approach starting distance. When analysed separately for the two most common species, common goldeneyes (*Bucephala clangula*) and mallards (*Anas platyrhynchos*), FID increased with increasing starting distance on rivers, but not lakes, likely because birds on lakes have enough time to evaluate the approaching object and take flight at great distance. Additionally, birds might perform different activities on lakes versus rivers, leading to varying energetic trade-offs between the two habitat types, which may affect the decision when to take flight. Finally, mallards aggregated in larger groups on lakes, which affected FID, likely due to enhanced visibility (i.e., earlier detection of the approaching boat) and detection probability (via increased group size) on lakes. Our results emphasize the importance of accounting for habitat characteristics, such as water body type, when studying waterbird FID, because they can affect the visibility of stimuli, group size and potentially animal behaviour, factors which should be taken into account when planning buffer zones for waterbirds in conservation areas.

## Introduction

Urbanisation and expanding infrastructure increasingly bring humans into closer contact with wildlife [1, 2]. Such interactions can stress and displace wildlife, changing individual behaviour, fitness, and ultimately population dynamics [3–5]. For example, recreational winter sports in remote alpine areas increase stress levels in black grouse (*Tetrao tetrix*) [6], and encroachment by eco-tourists reduces survival of Amazonian hoatzin birds (*Opisthocomus hoazin*) [7].

In many animals, approach by humans results in a flight response [8–11]. In birds, taking flight after human disturbance is costly and can reduce fitness [12], because of disruption to foraging opportunities [12–14], increased energy expenditure via increased flight duration [13, 15], and reduced ability to detect predators [16]. The impact of human disturbance on birds can be mediated by their flight initiation distance (the distance from a threat at which flight is initiated; hereafter FID). Hence, FID has become the focus of study in avian conservation biology, e.g. informing design of protective buffer zones against harmful effects of human presence [11].

Avian FID is a species-specific trait [17], and can depend on body size [18, 19] and individual body condition [20], with heavier birds and those in better condition taking flight at greater distances. Further, group size and hunting status (hunted versus protected species) can affect FID, with hunted species and larger groups being more vigilant, resulting in greater FID [18, 21]. Approach context, namely the distance at which an investigator’s approach begins (hereafter starting distance) [11], the number of approaching humans [22] and habitat type can all affect avian FID. For example, forest bird species alter FID dependent upon tree height and cover [23], but Fernández-Juricic, Venier [24] found little effect of vegetation structure grassland birds FID. More generally, individuals being in habitats with low food availability might have an increased FID due to reduced benefits of remaining in a resource poor area [25]. Despite numerous studies investigating bird FIDs in terrestrial environments by approaching them on foot [8, 17, 18, 26] and by car [27, 28], fewer studies have investigated impact of boats on FID in waterbirds, but see Ronconi and Clair [29] and Rodgers and Schwikert [30]. The finite nature of terrestrial water bodies (e.g., lakes and rivers) and their importance for waterbird foraging, migration and reproductive success, suggest that increasing levels of recreational boat traffic may severely impact some bird populations [31, 32].

**Fig 1:**
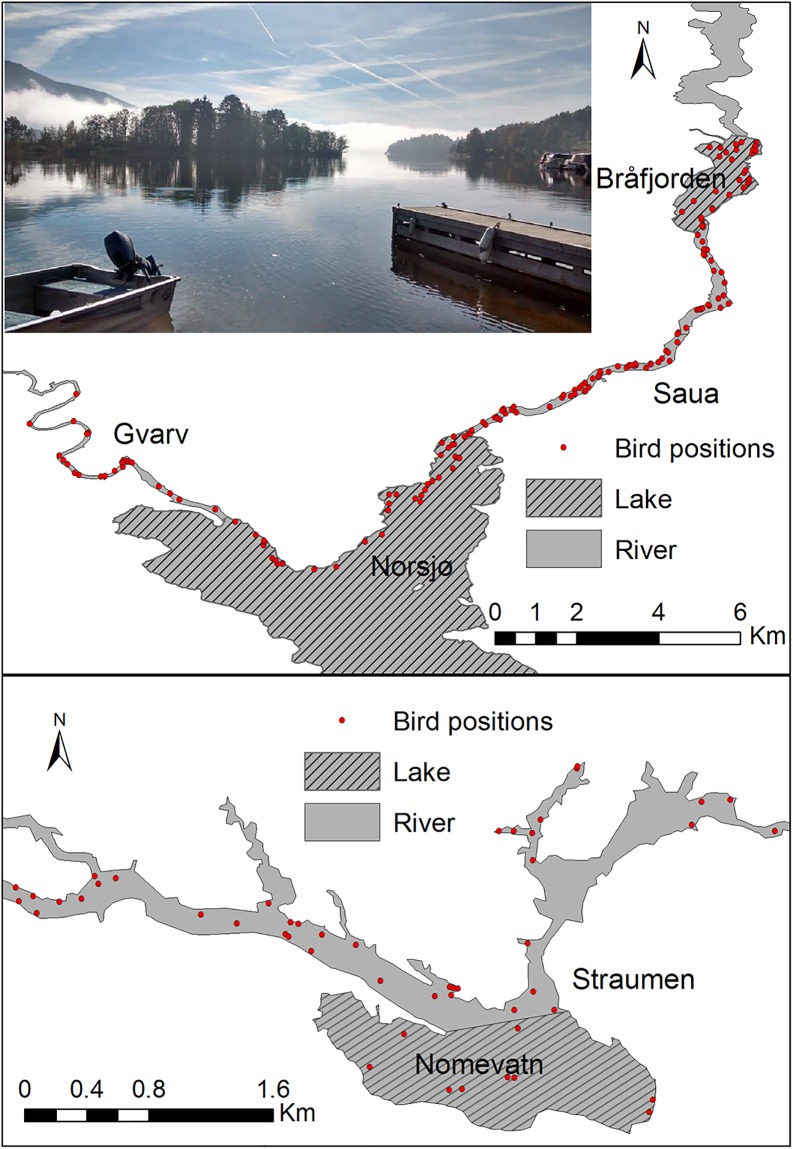
The study area in southeast Norway consisting of the rivers Gvarv, Saua and Straumen, and lakes Bråfjorden, Nomevatn and Norsjø. Red dots show the GPS positions of approached birds (group sizes are not indicated).

In this study, we examined FID of 10 Palearctic waterbird species in response to an approaching boat on two types of water bodies, i.e., lakes and rivers (Fig 1). Lakes represented more open habitat with a greater field of vision and thus starting distance, rivers more closed habitat. We predicted that 1a) water body type would influence FID, with birds taking flight earlier on lakes than rivers due to an increased approach starting distance and therefore earlier detection of approaches on lakes, and that 1b) water body type would affect bird group size, affecting FID. We predicted that 2) FID would be less in winter than in spring because birds are more energetically constrained during winter, 3) FID would be positively related to group size due to an increased detection probability of the approach, and 4) positively related to body mass, as larger bird species were assumed to require more time to initiate flight [30].

## Materials and methods

### Study area and bird approaches

Our study was conducted on three rivers (Gvarv, Saua, and Straumen) and three lakes (Norsjø, Bråfjorden, and Nomevatn) in Telemark County, southeast Norway (59°386′ N, 09°179′ E). The three rivers are between 50–200 m wide and occasionally open up and become natural lakes (about 500–3000 m wide, Fig 1). A mosaic of mixed-deciduous forest and crop fields surrounds the water bodies [33].

We approached waterbirds during daylight hours (between 0900 and 1500 hrs) using a 4 m long aluminium boat with a 9.9 horsepower outboard engine (approaches were conducted by one person; MM). Approaches were conducted only once per day and one to two times within a given season (spring or winter) to avoid repeatedly disturbing the same birds. We approached birds at 25 ± 5 km∙h^-1^, and used a handheld GPS unit (Garmin GPSmap 62s, Garmin Ltd) to record the location of the boat at the moment we observed the birds take flight. We then continued to the point from where birds took flight and recorded the flight location using the centre of the ripples created by the escaping birds (assumed to have an error of ±10 m due to GPS inaccuracy and water current). We calculated the FID as the Euclidean distance between the boat location (when we observed the birds taking flight) and the flight location in ArcMap 10.3 (Esri Redlands, CA). If birds did not fly before we were ≤ 10 m away, we abandoned the approach and recorded them as “not flying”, to avoid risk injury to birds. For each approach, we recorded water body type (river or lake), bird species, number of birds (group size), and estimated mean body mass (g) of each species from the literature (Table 1). To test if birds on lakes occurred farther from shore than on rivers, we calculated the perpendicular distance from the location of each bird (or group of birds using the centre position) to the shoreline. Data were collected in November and December 2015 and 2016 (hereafter winter; 146 approaches), and in April and May 2016 (hereafter spring; 69 approaches; Table 1). Finally, we calculated the ‘starting distance’ for each approach in ArcMap, measuring the straight-line distance from the bird location to the point at which the boat was first visible to the bird(s), i.e., the distance between the bird(s) and the boat, which was not intersected by any elevation (S1 Fig).

**Table 1:** Total number of observed individuals, average group size, average FID, and body mass for the nine bird species approached during this study in southeast Norway. Body mass was taken from the literature indicated in the last column.

Species	Approaches	Individuals	Average group size	FID in m (mean ± SD)	Bird mass (g)	Reference
Canada Goose (*Branta canadensis*)	17	275	16.2	183.7 ± 125.7	3,750	Mowbray, Ely [34]
Dipper (*Cinclus cinclus*)	11	11	1.0	26.9 ± 15.8	61	Bryant and Newton [35]
Eurasian Teal (*Anas crecca*)	4	51	12.8	168.6 ± 72.6	300	Guillemain, Elmberg [36]
Common Goldeneye (*Bucephala clangula*)	81	260	3.2	171.5 ± 86.9	656	Milonoff, Pöysä [37]
Goosander (*Mergus merganser*)	17	34	2.0	127.7 ± 73.5	1,872	Kalisińska, Budis [38]
Great Cormorant (*Phalacrocorax carbo*)	10	12	1.2	152.1 ± 56.9	2,380	Koffijberg and Vaneerden [39]
Heron (*Ardea cinerea*)	6	6	1.0	193.0 ± 57.6	1,600	Busse [40]
Mallard (*Anas platyrhynchos*)	34	272	8.0	92.2 ± 60.2	3,030	Girard and Grima [41]
Mute Swan (*Cygnus olor*)	30	60	2.0	119.4 ± 95.9	9,800	Beekman [42]
Whooper Swan (*Cygnus cygnus*)	5	23	4.6	211.4 ± 109.1	8,500	Pennycuick [43]

### Ethical statement

The Norwegian Experimental Animal Board (FOTS) granted a formal waiver of ethics approval for this study.

### Statistical analysis

We initially tested for differences in group size, distance of birds to the shore, and starting distance between lakes and rivers by using Mann-Whitney *U* tests to account for non-normality of the data. To identify general patterns, we modelled the FID of all bird species together (only birds that took flight were included in this analysis) by fitting Bayesian Markov Chain Monte Carlo phylogenetic generalised linear mixed models, using the R package ‘MCMCglmm’ [44], including species as a random effect. This approach was chosen to control for the phylogenetic relatedness of the species and the different sample size of FIDs across species [45]. We obtained phylogenetic data by downloading 100 trees from the Global Phylogeny of Birds website—www.birdtree.org [46], from which we calculated a 50% majority-rule consensus phylogeny using the R package ape [47]. We included group size (log-transformed to normalize residuals of the statistical models), species body mass, season (winter versus spring), water body type (lake versus river), starting distance (log-transformed), and the two-way interactions of starting distance x water body type and group size × water body type as fixed effects. The interactions were included to test whether group size and starting distance affected FID differently on rivers versus lakes. We scaled and centred the numeric fixed effects to increase convergence. For the Bayesian analyses, we used uninformative priors for fixed and random effects (variance = 1, nu = 0.002). Analyses were carried out with 1,000,000 iterations, with burn-in of 10,000 and thinning interval of 100. Furthermore, we modelled FID separately for the two most common species, the common goldeneye (*Bucephala clangula*, hereafter goldeneye, *n* = 81 approaches) and the mallard (*Anas platyrhynchos*, *n* = 34 approaches), using linear models with a Gaussian distribution and an identity link. Sample sizes for the other species were too small for separate analyses. For the goldeneye analysis, group size, starting distance, water body type, and the two-way interactions of starting distance x water body type and group size × water body type were included as independent variables. In the mallard analysis, group size and water body type were highly correlated (r = 0.723, p < 0.001). We therefore only included water body type, starting distance, and the interaction of starting distance x water body type as independent variables. Season was not included in the goldeneye and mallard analysis, because it was found to be uninformative in the analysis including all species (see below) and to avoid overfitting the models. All independent variables in all analyses had Pearson correlations < 0.6 and variance inflation factor values were < 3 [48]. We used the dredge function in R package MuMIn [49] to create a set of candidate models including all possible combinations of fixed effects and the above mentioned interactions. Model selection was based on Akaike’s Information Criterion for small sample sizes (AICc values), selecting the model with the lowest AICc value [50]. Parameters that included zero within their 95% confidence intervals (CI) were considered uninformative [51]. Data are shown as mean ± standard deviation (SD) unless otherwise stated. We validated the most parsimonious models by plotting the model residuals against the fitted values [48]. All analyses were performed in R 3.6.0 [52].

## Results

### General patterns

We conducted 215 approaches (146 on rivers and 69 on lakes) on 1,004 individuals of ten species (Table 1, S1 Table). Mean group size was 4.7 ± 8.4 birds (median = 2, range: 1–55 birds, Table 1), and group sizes were generally larger on lakes compared to rivers (7.6 ± 11.1 versus 3.3 ± 6.4, p < 0.001). When analysed separately, groups were not significantly larger on lakes compared to rivers (5.5 ± 6.1 individuals on lakes versus 2.5 ± 1.5 individuals on rivers, p = 0.080) for goldeneyes, but mallard groups were significantly larger on lakes than rivers (19.7 ± 14.4 versus 3.8 ± 7.1 individuals, p < 0.001). Birds approached on lakes were located significantly farther from the shore as compared to rivers (103 ± 84 m versus 30 ± 24 m, p < 0.001), and they were approached from a significantly greater starting distance (710 ± 387 m on lakes versus 465 ± 288 m on rivers, p < 0.001). Most birds (91%) took flight when approached by the boat, and the FID varied among species (ANOVA: F = 6.226, p < 0.001, Table 1). Mute swans (*Cygnus olor*) did not initiate flight in 47% (7 of 15 approaches) on lakes, and in 80% (12 of 15 approaches) on rivers. Their group size did not differ between rivers and lakes (p = 0.378), but swans were located further from the shore on lakes compared to rivers (p = 0.005). All individuals of other bird species took flight when approached.

### Flight initiation distance

**Fig 2:**
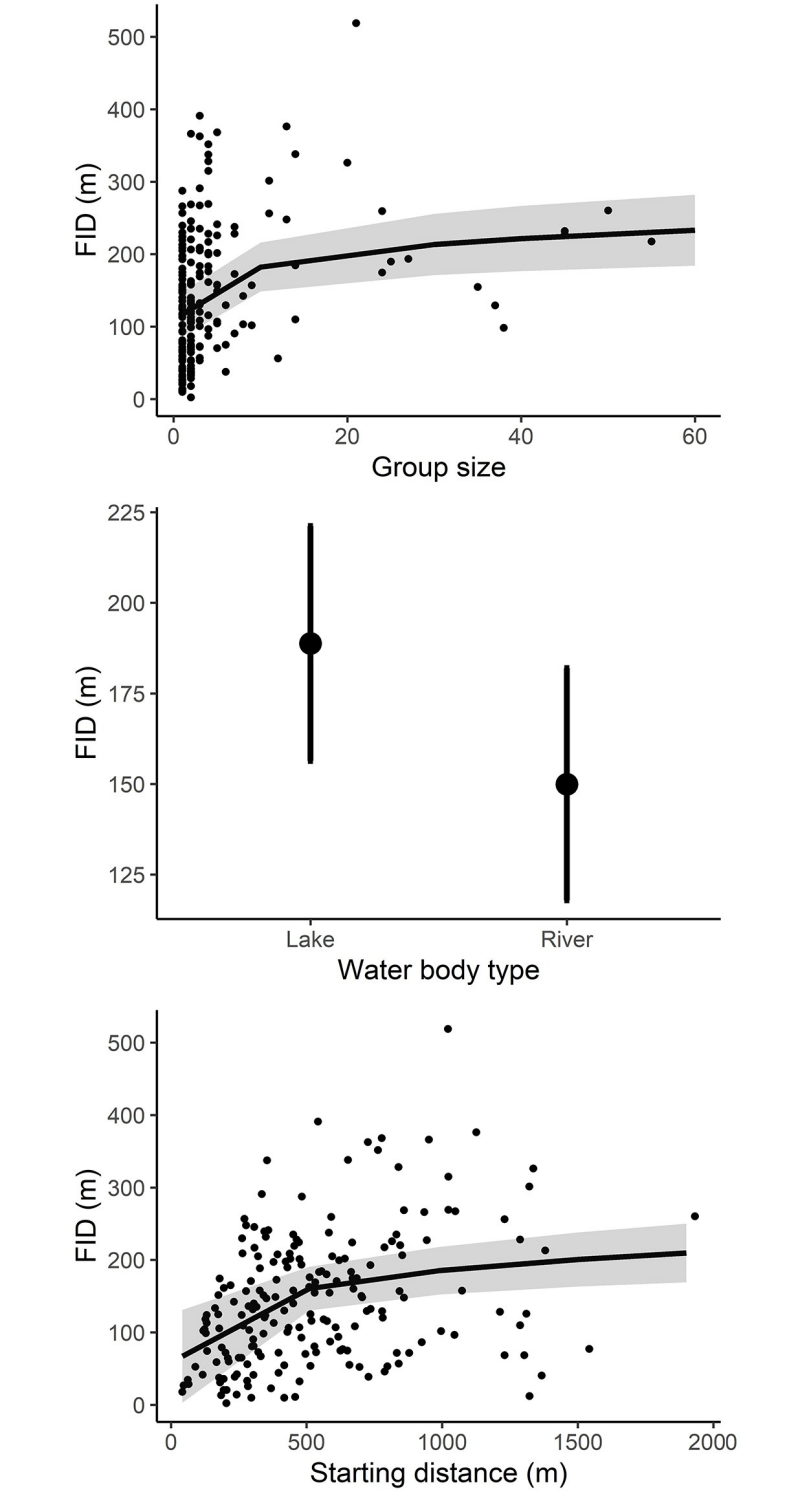
The predicted flight initiation distance (FID) of waterbirds (*n* = 196 approaches) in relation to the group size (top), the water body type (middle), and the starting distance (bottom) in southeast Norway in 2015 and 2016. Grey shading represents 95% confidence intervals.

**Fig 3:**
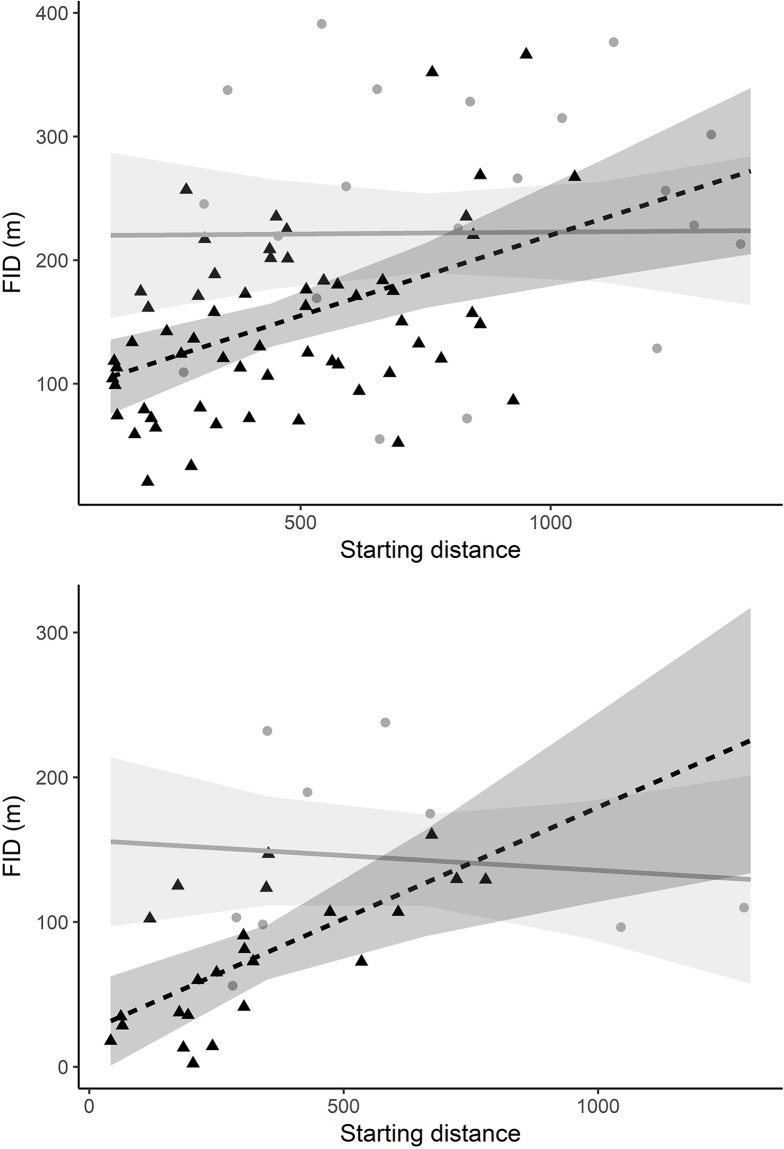
The predicted flight initiation distance (FID) of common goldeneyes (top) and mallards (bottom) in relation to the interaction of water body type and starting distance in southeast Norway in 2015 and 2016. Solid grey lines represent the predicted FID on lakes and dashed black lines the predicted FID on rivers. Raw data is shown with grey dots on lakes, and with black triangles on rivers. Grey shading represents 95% confidence intervals.

The FID of birds was on average 145 ± 92 m (range, 10–519 m). Variation in FID (all species analysed together) was best explained by group size, water body type, and starting distance (Table 2). Birds took flight earlier with increasing group size, when on lakes compared to rivers (FID = 192 ± 107 m versus 123 ± 75 m), and when approached from a longer starting distance (Fig 2). Species body mass, season and the interactions starting distance x water body type and group size × water body type were uninformative. The FID of goldeneyes (separate analysis) was larger on lakes compared to rivers and increased with increasing group size (Table 2). Moreover, the interaction between water body type and starting distance revealed that goldeneye FID was positively related to starting distance on rivers, but not lakes (Table 2, Fig 3).

**Table 2:** The best model explaining the flight initiation distance in European waterbirds approached from a boat in 2015 and 2016 in southeast Norway, separately for (1) all birds species together, (2) goldeneyes, and (3) mallards. β, estimated coefficient; SE, standard error; LCI, lower limit of the 95% confidence interval; UCI, upper limit of the 95% confidence interval; bold font indicates informative parameters.

Parameter	Estimate	SE	LCI	UCI	P
*All bird species*					
**Log (group size)**	**40.43**	**10.56**	**23.46**	**56.59**	**<0.001**
**Water body type (river)**	**-35.82**	**11.29**	**-60.57**	**-12.56**	**0.005**
**Starting distance**	**16.69**	**5.06**	**4.82**	**27.63**	**0.004**
Log (group size) x Water body type (river)	-21.23	12.56	-43.16	0.30	0.053
*Common goldeneye*					
**Log (group size)**	**48.39**	**16.10**	**16.49**	**80.30**	**0.003**
**Water body type (river)**	**-64.50**	**21.67**	**-107.66**	**-21.34**	**0.003**
Starting distance	1.80	14.71	-27.49	31.09	0.904
**Starting distance x Water body type (river)**	**41.77**	**18.96**	**4.00**	**79.54**	**0.030**
Log (group size) x Water body type (river)	-36.85	20.91	-78.51	4.82	0.083
*Mallard*					
Water body type (river)	-37.69	20.32	-79.19	3.80	0.073
Starting distance	-7.02	15.13	-37.91	23.87	0.646
**Starting distance x Water body type (river)**	**59.22**	**21.38**	**15.55**	**102.89**	**0.010**

## Discussion

We studied factors affecting European waterbird FIDs in response to approach by a boat and showed that birds generally initiated flight at greater distances on lakes compared to rivers, when group sizes were larger, and with longer approach starting distance.

Our results suggest that water body type, or more generally the habitat type, can directly and indirectly affect FID. Birds were located significantly further from the shore on lakes and were approached from a greater starting distance compared to birds on rivers. This likely allowed bird’s earlier detection of the approaching boat on lakes compared to rivers, directly affecting the FID. Similarly, the FID in Australian birds increased with increasing approach starting distances, indicating that birds may have had a longer period in which to detect the approach [8]. Spot-winged pigeons (*Columba maculosa*) and Mexican spotted owls (*Strix occidentalis lucida*) initiated flight earlier when perching on higher trees (likely due to greater visibility and thus approach starting distance) [23, 53], but in other species this relationship was reversed, possibly due to a reduced perception of risk [23]. In line with this latter hypothesis, being closer to shore (i.e., on rivers) could have given birds more concealment (e.g., via overhanging vegetation), which in effect could have led to a reduced FID due to a reduced perception of risk. For example, increasing vegetation concealment resulted in decreased FID in pygmy rabbits (*Brachylagus idahoensis*) [54]. Alternatively, energetic trade-offs might have differed between the two water body types. Many bird species utilize different habitats for different reasons, e.g. foraging and resting [55]. Consequently, birds might have different activity budgets on rivers and lakes, e.g. due to varying food availability [25]. It is plausible that birds spend more time foraging on rivers making it more costly to initiate flight, which could have led to the reduced FID there. Similarly, black ducks (*Anas rubripes*) rested more with increasing tide level, potentially due to reduced foraging opportunities [56]. Interestingly, we found that FID of goldeneyes and mallards was affected by the starting distance only on rivers, but not on lakes. The effect of starting distance likely levels off at longer starting distances (as seen on lakes) at which birds have enough time to evaluate the approaching object and take flight at great distance.

Further, group size affected FID. The analysis of all bird species pooled and the goldeneye analysis showed that birds increased their FID with increasing group size independent of water body type. Mallard flocked in larger groups on lakes compared to rivers and group sizes were positively related to the FID, so water body type potentially indirectly influenced mallard FID via group size. Larger group size facilitates earlier detection of potential threats [21, 57]. For example, roe deer (*Capreolus capreolus*) can exhibit an antipredator response by aggregation in larger groups in open habitats [58].

Mute swans remained on the water in 47% and 80% of approaches on lakes and rivers, respectively. Thus, the decision to initiate flight may relate to the starting distance, with swans being more likely to initiate flight when approached from a greater distance, i.e., on lakes, and as mentioned above swans might perform different activities on the two water bodies types rendering flight from rivers more costly. More generally, swans are a non-game species in all of Europe [59], and have been protected in large parts of Europe for almost a century [60]. We speculate that this long period of protection in a non-migratory species might have led to a habituation towards local human disturbance [61]. The other two large-bodied species in our study, whooper swans (*Cygnus cygnus*) and grey herons (*Ardea cinerea*), are non-hunted and migratory [62, 63], but herons—although being protected in Norway—are still legally hunted in parts of Europe, e.g. in Poland [64]. Generally, herons have experienced a long history of human persecution [65, 66], which might explain their greater FID.

### Conclusions

Our findings highlight that habitat, e.g., a water body, can affect avian FID in response to perceived threats (but see Fernández-Juricic, Venier [24]). Lakes represent an open habitat, resulting in larger starting distances of approaches compared to rivers. This likely allowed birds to detect approaches earlier on lakes than on rivers, which directly resulted in an increased FID. Additionally, resource availability might differ between habitats, resulting in different energetic trade-offs [25]. In the case of mallards, the water body type also indirectly altered the bird FID via altered group sizes, with mallards aggregating in larger groups on lakes compared to rivers. Buffer zone distances for waterbirds in conservation areas are based on FID studies [30, 67, 68], but these do not take into account the variability of FID according to habitat type, e.g., water body type as in our study. To provide more conservative buffer zones that ensure the proper protection of waterbird species, managers should take this variability into account. Specifically, open habitats where animals can perceive a disturbance from a greater distance (e.g. lakes versus rivers, or grassland versus forest) and habitats that are more important for specific activities, such as breeding and foraging, should have greater buffer zones to avoid negative impacts of disturbance.

## Supporting information

S1 Fig Illustration of how we calculated the starting distance: The starting distance was defined as the maximum straight-line distance between the boat and the bird location not intersected by any elevation or vegetation. Source of digital elevation model: https://kartkatalog.geonorge.no.
(DOCX)

S1 Table All raw data of our water bird approaches. (DOCX)
