# Supplementary material for: Water body type and group size affect the flight initiation distance of European waterbirds
Source: PLoS One. 2019 Jul 16;14(7):e0219845. doi: 10.1371/journal.pone.0219845 (PMC6634859; doi:10.1371/journal.pone.0219845)
Supplement: S1 Fig — Source of digital elevation model: https://kartkatalog.geonorge.no. (DOCX) [file pone.0219845.s001.docx]

**Supporting information**


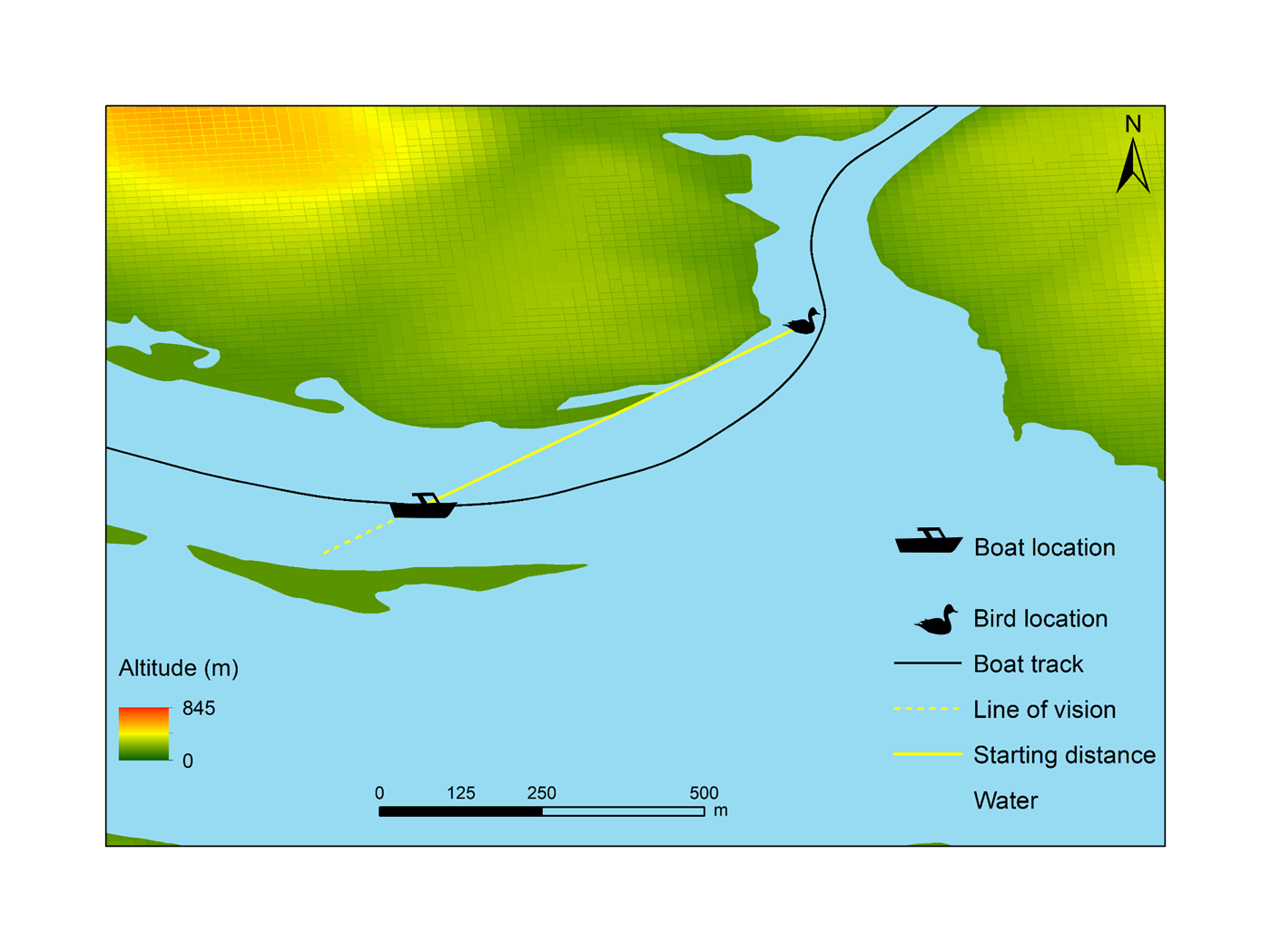


**S1 Fig.** Illustration of how we calculated the starting distance: the starting distance was defined as the maximum straight-line distance between the boat and the bird location not intersected by any elevation or vegetation. Source of digital elevation model: https://kartkatalog.geonorge.no
