# Supplementary material for: Water body type and group size affect the flight initiation distance of European waterbirds
Source: PLoS One. 2019 Jul 16;14(7):e0219845. doi: 10.1371/journal.pone.0219845 (PMC6634859; doi:10.1371/journal.pone.0219845)
Supplement: S1 Table — (DOCX) [file pone.0219845.s002.docx]

**Supporting information**

**S1 Table.** All raw data of our water bird approaches.

| Approach | Species | Groupsize | Distfromshore | Flight | FID | Mass | Type | Starting_distance |
| --- | --- | --- | --- | --- | --- | --- | --- | --- |
| 1 | Mute Swan | 1 | 13.58 | 0 | NA | 9800 | River | 565 |
| 2 | Goldeneye | 3 | 61.63 | 1 | 130.03 | 656 | River | 418 |
| 3 | Mute Swan | 2 | 44.98 | 0 | NA | 9800 | River | 545 |
| 4 | Mute Swan | 1 | 19.67 | 0 | NA | 9800 | River | 580 |
| 5 | Heron | 1 | 4.52 | 1 | 287.39 | 1600 | River | 483 |
| 6 | Goldeneye | 7 | 47.67 | 1 | 172.7 | 656 | River | 389 |
| 7 | Heron | 1 | 1.97 | 1 | 224.08 | 1600 | River | 668 |
| 8 | Goldeneye | 1 | 55.17 | 1 | 150.33 | 656 | River | 702 |
| 9 | Goosander | 5 | 66.97 | 1 | 149.05 | 1872 | River | 385 |
| 10 | Mute Swan | 3 | 81.54 | 1 | 362.61 | 9800 | Lake | 726 |
| 11 | Cormorant | 2 | 237.24 | 1 | 206.49 | 2380 | Lake | 853 |
| 12 | Goldeneye | 13 | 110.68 | 1 | 376.24 | 656 | Lake | 1126 |
| 13 | Canada Goose | 21 | 92.8 | 1 | 518.99 | 3750 | Lake | 1021 |
| 14 | Canada Goose | 50 | 147.29 | 1 | 260.16 | 3750 | Lake | 1932 |
| 15 | Mallard | 24 | 37.08 | 1 | 174.8 | 3030 | Lake | 670 |
| 16 | Mute Swan | 3 | 46.32 | 1 | 100.58 | 9800 | Lake | 429 |
| 17 | Mute Swan | 2 | 21.78 | 1 | 32.25 | 9800 | Lake | 474 |
| 18 | Mute Swan | 1 | 135.93 | 0 | NA | 9800 | Lake | 263 |
| 19 | Canada Goose | 20 | 199.4 | 1 | 326.33 | 3750 | Lake | 1336 |
| 20 | Goldeneye | 14 | 74.03 | 1 | 338.2 | 656 | Lake | 653 |
| 21 | Dipper | 1 | 9.87 | 1 | 40.31 | 61 | River | 1367 |
| 22 | Dipper | 1 | 4.03 | 1 | 12.08 | 61 | River | 1322 |
| 23 | Dipper | 1 | 3.24 | 1 | 22.83 | 61 | River | 369 |
| 24 | Canada Goose | 1 | 27.02 | 1 | 229.94 | 3750 | River | 262 |
| 25 | Goldeneye | 5 | 55.97 | 1 | 157.06 | 656 | River | 843 |
| 26 | Goosander | 1 | 21.98 | 1 | 25.55 | 1872 | River | 284 |
| 27 | Canada Goose | 9 | 198.46 | 1 | 157.14 | 3750 | Lake | 277 |
| 28 | Goldeneye | 4 | 253.1 | 1 | 337.52 | 656 | Lake | 354 |
| 29 | Mute Swan | 3 | 242.86 | 1 | 205 | 9800 | Lake | 321 |
| 30 | Mute Swan | 4 | 257.43 | 0 | NA | 9800 | Lake | 572 |
| 31 | Dipper | 1 | 8.05 | 1 | 30.81 | 61 | Lake | 181 |
| 32 | Mallard | 7 | 168.59 | 1 | 237.76 | 3030 | Lake | 582 |
| 33 | Canada Goose | 1 | 299.55 | 1 | 65 | 3750 | Lake | 260 |
| 34 | Heron | 1 | 30.62 | 1 | 205.02 | 1600 | River | 595 |
| 35 | Goldeneye | 1 | 13.88 | 1 | 219.46 | 656 | Lake | 455 |
| 36 | Goosander | 1 | 18.11 | 1 | 38.6 | 1872 | Lake | 235 |
| 37 | Goosander | 1 | 155.31 | 1 | 197.89 | 1872 | Lake | 423 |
| 38 | Heron | 1 | 126.95 | 1 | 154.57 | 1600 | Lake | 585 |
| 39 | Goldeneye | 1 | 33.13 | 1 | 71.81 | 656 | Lake | 833 |
| 40 | Goldeneye | 1 | 105.51 | 1 | 213 | 656 | Lake | 1380 |
| 41 | Cormorant | 1 | 52.6 | 1 | 125.92 | 2380 | Lake | 1310 |
| 42 | Goldeneye | 2 | 21.27 | 1 | 109.2 | 656 | Lake | 266 |
| 43 | Goldeneye | 3 | 68.69 | 1 | 169.12 | 656 | Lake | 532 |
| 44 | Goldeneye | 5 | 73.24 | 1 | 225.8 | 656 | Lake | 815 |
| 45 | Mute Swan | 6 | 44.42 | 0 | NA | 9800 | River | 474 |
| 46 | Mallard | 2 | 38.46 | 1 | 37.48 | 3030 | River | 177 |
| 47 | Goldeneye | 3 | 58.66 | 1 | 174.52 | 656 | River | 180 |
| 48 | Goosander | 2 | 25.6 | 1 | 105.55 | 1872 | River | 178 |
| 49 | Goldeneye | 5 | 14.43 | 1 | 104.31 | 656 | River | 124 |
| 50 | Goosander | 3 | 30.12 | 1 | 209.12 | 1872 | River | 264 |
| 51 | Goldeneye | 8 | 20.46 | 1 | 142.28 | 656 | River | 232 |
| 52 | Goldeneye | 2 | 25.97 | 1 | 120.57 | 656 | River | 345 |
| 53 | Goosander | 1 | 21.51 | 1 | 151.65 | 1872 | River | 175 |
| 54 | Mallard | 3 | 21.9 | 1 | 72.78 | 3030 | River | 322 |
| 55 | Dipper | 1 | 5.02 | 1 | 41.59 | 61 | River | 117 |
| 56 | Goldeneye | 2 | 13.07 | 1 | 133.45 | 656 | River | 163 |
| 57 | Mute Swan | 2 | 19.11 | 1 | 139.98 | 9800 | River | 306 |
| 58 | Mute Swan | 4 | 19.11 | 0 | NA | 9800 | River | 187 |
| 59 | Goldeneye | 1 | 16.74 | 1 | 79.23 | 656 | River | 187 |
| 60 | Dipper | 1 | 4.42 | 1 | 52.48 | 61 | River | 90 |
| 61 | Goosander | 1 | 27.13 | 1 | 165.15 | 1872 | River | 220 |
| 62 | Goldeneye | 2 | 23.62 | 1 | 124.15 | 656 | River | 261 |
| 63 | Goosander | 1 | 14.38 | 1 | 123.98 | 1872 | Lake | 132 |
| 64 | Cormorant | 1 | 56.36 | 1 | 239.45 | 2380 | Lake | 344 |
| 65 | Goldeneye | 1 | 49.69 | 1 | 174.9 | 656 | River | 685 |
| 66 | Canada Goose | 14 | 55.31 | 1 | 184.5 | 3750 | River | 555 |
| 67 | Canada Goose | 35 | 49.86 | 1 | 154.62 | 3750 | River | 530 |
| 68 | Mute Swan | 1 | 21.56 | 0 | NA | 9800 | River | 558 |
| 69 | Whooper Swan | 4 | 13.69 | 1 | 87.32 | 8500 | River | 587 |
| 70 | Mute Swan | 2 | 63.37 | 0 | NA | 9800 | River | 604 |
| 71 | Goldeneye | 5 | 62.84 | 1 | 201.44 | 656 | River | 440 |
| 72 | Goldeneye | 1 | 30.77 | 1 | 148.14 | 656 | River | 859 |
| 73 | Goldeneye | 1 | 7.21 | 1 | 80.66 | 656 | River | 299 |
| 74 | Goldeneye | 2 | 33.94 | 1 | 268.61 | 656 | River | 859 |
| 75 | Goosander | 3 | 56.83 | 1 | 207.65 | 1872 | River | 393 |
| 76 | Heron | 1 | 8.2 | 1 | 151.2 | 1600 | River | 341 |
| 77 | Dipper | 1 | 5.26 | 1 | 44.18 | 61 | River | 397 |
| 78 | Canada Goose | 4 | 37.34 | 1 | 199.45 | 3750 | Lake | 620 |
| 79 | Mute Swan | 2 | 59.6 | 0 | NA | 9800 | Lake | 268 |
| 80 | Canada Goose | 45 | 57.17 | 1 | 227.38 | 3750 | Lake | 943 |
| 81 | Mute Swan | 2 | 81.31 | 0 | NA | 9800 | Lake | 1086 |
| 82 | Mute Swan | 1 | 169.1 | 0 | NA | 9800 | Lake | 1013 |
| 83 | Whooper Swan | 5 | 53.75 | 1 | 241.29 | 8500 | River | 359 |
| 84 | Goldeneye | 1 | 13.25 | 1 | 67.01 | 656 | River | 331 |
| 85 | Dipper | 1 | 39.06 | 1 | 9.9 | 61 | River | 296 |
| 86 | Mute Swan | 2 | 37.81 | 0 | NA | 9800 | River | 479 |
| 87 | Goldeneye | 4 | 62.01 | 1 | 176.14 | 656 | River | 511 |
| 88 | Canada Goose | 55 | 30.89 | 1 | 217.55 | 3750 | River | 787 |
| 89 | Cormorant | 1 | 36.5 | 1 | 157.71 | 2380 | River | 1073 |
| 90 | Goosander | 1 | 111 | 1 | 77.13 | 1872 | River | 1542 |
| 91 | Mallard | 2 | 7.77 | 1 | 35.69 | 3030 | River | 194 |
| 92 | Cormorant | 1 | 16.64 | 1 | 197.14 | 2380 | River | 378 |
| 93 | Goldeneye | 1 | 21.81 | 1 | 59.03 | 656 | River | 168 |
| 94 | Mute Swan | 1 | 12.45 | 0 | NA | 9800 | River | 1204 |
| 95 | Cormorant | 1 | 56.14 | 1 | 77.03 | 2380 | River | 633 |
| 96 | Dipper | 1 | 2.12 | 1 | 20.62 | 61 | River | 205 |
| 97 | Mallard | 2 | 2.88 | 1 | 2.24 | 3030 | River | 204 |
| 98 | Goldeneye | 1 | 18.97 | 1 | 20.59 | 656 | River | 194 |
| 99 | Mallard | 2 | 7.28 | 1 | 17.89 | 3030 | River | 42 |
| 100 | Goldeneye | 3 | 41.25 | 1 | 108.47 | 656 | River | 678 |
| 101 | Mallard | 6 | 29.51 | 1 | 129.62 | 3030 | River | 722 |
| 102 | Mallard | 2 | 10.41 | 1 | 72.56 | 3030 | River | 535 |
| 103 | Canada Goose | 2 | 18.6 | 1 | 38.59 | 3750 | River | 728 |
| 104 | Mallard | 6 | 14.55 | 1 | 37.59 | 3030 | River | 177 |
| 105 | Mallard | 2 | 21.28 | 1 | 106.93 | 3030 | River | 473 |
| 106 | Goldeneye | 4 | 16.08 | 1 | 183.38 | 656 | River | 546 |
| 107 | Goldeneye | 2 | 24.21 | 1 | 86.35 | 656 | River | 925 |
| 108 | Mallard | 2 | 6.96 | 1 | 123.57 | 3030 | River | 348 |
| 109 | Goldeneye | 3 | 36.31 | 1 | 132.38 | 656 | River | 736 |
| 110 | Mallard | 2 | 61.36 | 1 | 160.22 | 3030 | River | 673 |
| 111 | Mute Swan | 1 | 35.27 | 1 | 92.7 | 9800 | River | 481 |
| 112 | Goldeneye | 2 | 48.88 | 1 | 235.13 | 656 | River | 451 |
| 113 | Mallard | 2 | 2.87 | 1 | 65.12 | 3030 | River | 250 |
| 114 | Mute Swan | 1 | 20.47 | 0 | NA | 9800 | River | 293 |
| 115 | Goldeneye | 1 | 28.45 | 1 | 170.94 | 656 | River | 295 |
| 116 | Goldeneye | 3 | 18.4 | 1 | 183.58 | 656 | River | 664 |
| 117 | Mallard | 37 | 10.55 | 1 | 129.2 | 3030 | River | 779 |
| 118 | Canada Goose | 3 | 22.13 | 1 | 53.26 | 3750 | River | 798 |
| 119 | Goldeneye | 2 | 11.24 | 1 | 98.79 | 656 | River | 129 |
| 120 | Goldeneye | 2 | 9.66 | 1 | 162.69 | 656 | River | 510 |
| 121 | Mallard | 1 | 30.56 | 1 | 81.15 | 3030 | River | 305 |
| 122 | Goldeneye | 1 | 38.96 | 1 | 180.14 | 656 | River | 574 |
| 123 | Goldeneye | 4 | 67.46 | 1 | 201.19 | 656 | River | 474 |
| 124 | Goosander | 2 | 51.89 | 1 | 80.71 | 1872 | River | 529 |
| 125 | Eurasian Teal | 6 | 100.91 | 1 | 74.97 | 300 | River | 650 |
| 126 | Goldeneye | 2 | 19.1 | 1 | 125.1 | 656 | River | 514 |
| 127 | Goldeneye | 2 | 36.44 | 1 | 113 | 656 | River | 379 |
| 128 | Mallard | 2 | 12.81 | 1 | 41.34 | 3030 | River | 304 |
| 129 | Canada Goose | 2 | 9.64 | 1 | 42.05 | 3750 | River | 243 |
| 130 | Goldeneye | 1 | 26.86 | 1 | 208.73 | 656 | River | 438 |
| 131 | Mallard | 1 | 17.58 | 1 | 147 | 3030 | River | 352 |
| 132 | Goldeneye | 3 | 70.43 | 1 | 235.17 | 656 | River | 831 |
| 133 | Goosander | 1 | 0.9 | 1 | 68.36 | 1872 | River | 1230 |
| 134 | Goldeneye | 1 | 32.09 | 1 | 118.02 | 656 | River | 563 |
| 135 | Goldeneye | 2 | 19.88 | 1 | 118.34 | 656 | River | 127 |
| 136 | Mallard | 2 | 7.48 | 1 | 34.71 | 3030 | River | 62 |
| 137 | Mallard | 1 | 23.35 | 1 | 125.02 | 3030 | River | 174 |
| 138 | Goldeneye | 2 | 23.81 | 1 | 157.8 | 656 | River | 327 |
| 139 | Goldeneye | 2 | 8.56 | 1 | 220.27 | 656 | River | 845 |
| 140 | Goldeneye | 4 | 16.97 | 1 | 217.01 | 656 | River | 308 |
| 141 | Goosander | 1 | 9.18 | 1 | 26.93 | 1872 | River | 47 |
| 142 | Mallard | 2 | 4.12 | 1 | 28.44 | 3030 | River | 65 |
| 143 | Goldeneye | 1 | 49.09 | 1 | 256.81 | 656 | River | 271 |
| 144 | Goldeneye | 3 | 30.6 | 1 | 72.11 | 656 | River | 201 |
| 145 | Mallard | 1 | 9.94 | 1 | 102.31 | 3030 | River | 119 |
| 146 | Goldeneye | 4 | 32.75 | 1 | 161.38 | 656 | River | 195 |
| 147 | Mallard | 1 | 1.08 | 1 | 14.21 | 3030 | River | 242 |
| 148 | Goldeneye | 2 | 11.52 | 1 | 64.4 | 656 | River | 210 |
| 149 | Mallard | 1 | 0.21 | 1 | 13.15 | 3030 | River | 185 |
| 150 | Canada Goose | 3 | 20.13 | 1 | 290.87 | 3750 | River | 335 |
| 151 | Mallard | 5 | 63.54 | 1 | 106.89 | 3030 | River | 607 |
| 152 | Canada Goose | 1 | 14.98 | 1 | 54.63 | 3750 | River | 418 |
| 153 | Mallard | 1 | 6.92 | 1 | 59.77 | 3030 | River | 213 |
| 154 | Goldeneye | 2 | 10.78 | 1 | 74.33 | 656 | River | 133 |
| 155 | Canada Goose | 9 | 5.63 | 1 | 101.91 | 3750 | Lake | 996 |
| 156 | Mute Swan | 1 | 10.43 | 0 | NA | 9800 | Lake | 1041 |
| 157 | Goosander | 2 | 229.08 | 1 | 71.57 | 1872 | Lake | 878 |
| 158 | Goldeneye | 1 | 205.94 | 1 | 266.06 | 656 | Lake | 934 |
| 159 | Goldeneye | 11 | 130.35 | 1 | 256.19 | 656 | Lake | 1230 |
| 160 | Goldeneye | 4 | 95.9 | 1 | 228.09 | 656 | Lake | 1287 |
| 161 | Mute Swan | 1 | 62.62 | 0 | NA | 9800 | Lake | 1506 |
| 162 | Goldeneye | 2 | 87.21 | 1 | 245.46 | 656 | Lake | 307 |
| 163 | Goldeneye | 2 | 66.97 | 1 | 136.24 | 656 | River | 286 |
| 164 | Goldeneye | 3 | 24.92 | 1 | 267.23 | 656 | River | 1048 |
| 165 | Goldeneye | 2 | 37.18 | 1 | 188.48 | 656 | River | 328 |
| 166 | Mute Swan | 1 | 173.289394 | 0 | NA | 9800 | River | 217 |
| 167 | Mallard | 7 | 17.510575 | 1 | 90.60905032 | 3030 | River | 303 |
| 168 | Goldeneye | 2 | 53.069522 | 1 | 106.3202709 | 656 | River | 434 |
| 169 | Goldeneye | 2 | 55.704053 | 1 | 72.00694411 | 656 | River | 397 |
| 170 | Goldeneye | 1 | 58.116022 | 1 | 170.8361788 | 656 | River | 611 |
| 171 | Goldeneye | 2 | 33.719593 | 1 | 52.20153254 | 656 | River | 695 |
| 172 | Goldeneye | 3 | 31.815607 | 1 | 120.2081528 | 656 | River | 781 |
| 173 | Dipper | 1 | 6.852211 | 1 | 9.899494937 | 61 | River | 418 |
| 174 | Goldeneye | 5 | 26.549691 | 1 | 70.17834424 | 656 | River | 496 |
| 175 | Mute Swan | 2 | 32.301383 | 1 | 74.88658091 | 9800 | River | 625 |
| 176 | Mute Swan | 2 | 22.608186 | 0 | NA | 9800 | River | 582 |
| 177 | Goldeneye | 1 | 11.75839 | 1 | 94.04786016 | 656 | River | 617 |
| 178 | Eurasian Teal | 5 | 47.84632 | 1 | 157.800507 | 300 | River | 451 |
| 179 | Goldeneye | 4 | 27.048797 | 1 | 351.8081864 | 656 | River | 763 |
| 180 | Dipper | 1 | 23.769833 | 1 | 10.81665383 | 61 | River | 458 |
| 181 | Cormorant | 1 | 23.375724 | 1 | 193.0025907 | 2380 | River | 735 |
| 182 | Goldeneye | 2 | 32.781098 | 1 | 366.1966685 | 656 | River | 951 |
| 183 | Cormorant | 2 | 31.651786 | 1 | 68.42514158 | 2380 | River | 1303 |
| 184 | Goosander | 4 | 78.661263 | 1 | 201.7919721 | 1872 | Lake | 641 |
| 185 | Mute Swan | 3 | 20.122302 | 1 | 57.07013229 | 9800 | Lake | 840 |
| 186 | Eurasian Teal | 13 | 50.061645 | 1 | 247.9516082 | 300 | Lake | 277 |
| 187 | Goldeneye | 1 | 50.661592 | 1 | 55.21775077 | 656 | Lake | 658 |
| 188 | Goldeneye | 24 | 99.762786 | 1 | 259.5091521 | 656 | Lake | 591 |
| 189 | Goldeneye | 1 | 62.701144 | 1 | 128.4562182 | 656 | Lake | 1213 |
| 190 | Mallard | 14 | 17.025308 | 1 | 109.8954048 | 3030 | Lake | 1287 |
| 191 | Goldeneye | 11 | 107.447345 | 1 | 301.4962686 | 656 | Lake | 1321 |
| 192 | Mute Swan | 2 | 203.687822 | 1 | 53.75872022 | 9800 | Lake | 514 |
| 193 | Cormorant | 1 | 229.624824 | 1 | 115.8792475 | 2380 | Lake | 518 |
| 194 | Whooper Swan | 7 | 75.516329 | 1 | 228.0898946 | 8500 | Lake | 463 |
| 195 | Mallard | 38 | 92.844409 | 1 | 98.23441352 | 3030 | Lake | 341 |
| 196 | Mallard | 8 | 57.873588 | 1 | 103.0970417 | 3030 | Lake | 289 |
| 197 | Mallard | 12 | 122.260895 | 1 | 55.97320788 | 3030 | Lake | 282 |
| 198 | Whooper Swan | 2 | 93.119731 | 1 | 131.7459677 | 8500 | Lake | 302 |
| 199 | Mute Swan | 2 | 60.375444 | 1 | 46.01086828 | 9800 | Lake | 788 |
| 200 | Goldeneye | 3 | 266.423947 | 1 | 391.0511475 | 656 | Lake | 542 |
| 201 | Mute Swan | 1 | 404.943659 | 1 | 148.4082208 | 9800 | Lake | 705 |
| 202 | Goldeneye | 4 | 69.841729 | 1 | 314.8396417 | 656 | Lake | 1023 |
| 203 | Goosander | 4 | 69.841729 | 1 | 269.267525 | 1872 | Lake | 1023 |
| 204 | Cormorant | 1 | 43.199951 | 1 | 139.917833 | 2380 | Lake | 451 |
| 205 | Mallard | 45 | 92.547086 | 1 | 231.9353358 | 3030 | Lake | 350 |
| 206 | Mallard | 25 | 61.534454 | 1 | 189.6127633 | 3030 | Lake | 429 |
| 207 | Whooper Swan | 5 | 33.039037 | 1 | 368.3313725 | 8500 | Lake | 777 |
| 208 | Goldeneye | 4 | 22.756474 | 1 | 328.239242 | 656 | Lake | 839 |
| 209 | Mallard | 4 | 29.728349 | 1 | 96.42095208 | 3030 | Lake | 1045 |
| 210 | Heron | 1 | 53.478512 | 1 | 135.3513945 | 1600 | River | 316 |
| 211 | Eurasian Teal | 27 | 56.965895 | 1 | 193.494186 | 300 | River | 481 |
| 212 | Goldeneye | 1 | 36.38097 | 1 | 224.5551157 | 656 | River | 472 |
| 213 | Goldeneye | 4 | 37.746999 | 1 | 115.5205609 | 656 | River | 575 |
| 214 | Goldeneye | 1 | 7.436522 | 1 | 113.1591799 | 656 | River | 132 |
| 215 | Goldeneye | 1 | 12.810129 | 1 | 33.30165161 | 656 | River | 281 |
